# Mixed Chlamydia trachomatis Peptide Antigens Provide a Specific and Sensitive Single-Well Colorimetric Enzyme-Linked Immunosorbent Assay for Detection of Human Anti*-*C. trachomatis Antibodies

**DOI:** 10.1128/mSphere.00484-18

**Published:** 2018-11-07

**Authors:** K. Shamsur Rahman, Toni Darville, Harold C. Wiesenfeld, Sharon L. Hillier, Bernhard Kaltenboeck

**Affiliations:** a Department of Pathobiology, College of Veterinary Medicine, Auburn University, Auburn, Alabama, USA; b Department of Pediatrics, University of North Carolina at Chapel Hill, Chapel Hill, North Carolina, USA; c Department of Obstetrics, Gynecology and Reproductive Sciences, University of Pittsburgh School of Medicine and Magee-Womens Research Institute, Pittsburgh, Pennsylvania, USA; UMKC School of Medicine

**Keywords:** *Chlamydia trachomatis*, diagnosis, serology, antibody detection, B-cell epitopes, peptide antigens, ELISA, cross-reactivity, species-specific, antibody isotypes, IgG, IgG1, IgG3, IgA, IgA1, IgA2

## Abstract

Sensitive and specific detection of anti-Chlamydia trachomatis antibodies in standard enzyme-linked immunosorbent assays (ELISAs) is compromised by cross-reactivity and poor sensitivity of classical C. trachomatis antigens. Previously, we discovered 48 strongly reactive peptide antigens of C. trachomatis-specific B-cell epitopes from 21 immunodominant proteins. By comprehensive individual testing of 11 top-ranked peptide antigens, we found very high sensitivity and specificity for detection of anti-C. trachomatis antibodies in chemiluminescent ELISAs. The current study established a labor-saving colorimetric ELISA by using a mixture of 12 strongly reactive C. trachomatis peptide antigens (Ctr Mix1) in a single well/serum rather than assaying reactivity to each individual peptide. For performance evaluation, we used a simulated population of 212 anti-C. trachomatis antibody-positive and -negative sera from 125 women with NAAT-confirmed active C. trachomatis infection and from 87 healthy women at low risk for C. trachomatis infection. In comparison to a composite reference standard (CRS) for anti-C. trachomatis antibody status, the Ctr Mix1 IgG ELISA achieved 93.9% sensitivity, significantly superior to the 49% to 79% sensitivities of four commercial anti-C. trachomatis IgG ELISAs, and 98% specificity of all tested assays. Compared to the labor-intensive individual peptide testing, this mixed peptide ELISA retained high specificity with only marginal, ∼5% sensitivity loss. By ROC-AUC, likelihood ratio, and predictive value analyses, the Ctr Mix1 ELISA performed satisfactorily at 10% to 75% prevalence range of anti-C. trachomatis antibodies but significantly better than commercial ELISAs. Thus, the labor-saving mixed peptide colorimetric ELISA format provides simultaneously high specificity and sensitivity for detection of anti-C. trachomatis antibodies.

**IMPORTANCE** For detection of anti-C. trachomatis antibodies by serological assays, use of classical chlamydial antigens results in high cross-reactivity and poor sensitivity. Previously, we discovered 48 strongly reactive peptide antigens of C. trachomatis-specific B-cell epitopes from 21 immunodominant proteins, and individual testing and combined scoring of 5 to 11 peptide antigens provided highly sensitive and specific detection of anti-C. trachomatis antibodies in chemiluminescent ELISAs. To simplify this method, this study established a single-well labor-saving colorimetric ELISA using a mixture of 12 strongly reactive C. trachomatis peptide antigens (Ctr Mix1) for detection of anti-C. trachomatis antibodies. This Ctr Mix1 ELISA (94% sensitivity and 98% specificity) outperformed 4 commercial ELISAs (49% to 79% sensitivity and 98% specificity). This ELISA can be easily implemented and commercialized, with convenient setup for use in nonspecialized laboratories. Thus, this mixed peptide assay with superior specificity and sensitivity will improve serodiagnosis of C. trachomatis infections.

## Abstract (precis)

For detection of anti-C. trachomatis antibodies by serological assays, use of classical chlamydial antigens results in high cross-reactivity and poor sensitivity. Previously, we discovered 48 strongly reactive peptide antigens of C. trachomatis-specific B-cell epitopes from 21 immunodominant proteins, and individual testing and combined scoring of 5 to 11 peptide antigens provided highly sensitive and specific detection of anti-C. trachomatis antibodies in chemiluminescent ELISAs. To simplify this method, this study established a single-well labor-saving colorimetric ELISA using a mixture of 12 strongly reactive C. trachomatis peptide antigens (Ctr Mix1) for detection of anti-C. trachomatis antibodies. This Ctr Mix1 ELISA (94% sensitivity and 98% specificity) outperformed 4 commercial ELISAs (49% to 79% sensitivity and 98% specificity). This ELISA can be easily implemented and commercialized, with convenient setup for use in nonspecialized laboratories. Thus, this mixed peptide assay with superior specificity and sensitivity will improve serodiagnosis of C. trachomatis infections.

## INTRODUCTION

Current serological assays for species-specific detection of anti-Chlamydia trachomatis antibodies suffer from poor assay specificity due to cross-reactivity of conventional C. trachomatis antigens (1–12). Anti-C. trachomatis and anti-Chlamydia pneumoniae antibodies are highly prevalent in human populations (13–15). Therefore, novel specific assays, particularly in simple format such as enzyme-linked immunosorbent assay (ELISA), are urgently needed for human chlamydial serology.

Previously, we identified highly reactive and specific B-cell epitopes of immunodominant proteins of all *Chlamydia* species (16–21). An important finding was that 16- to 30-amino-acid peptide antigens combined with an N-terminal hydrophilic serine-glycine-serine-glycine spacer were required for optimal signal strength (16–18). Further testing of predicted peptide antigens with sera from C. trachomatis*-*infected women revealed additional human host-specific C. trachomatis B-cell epitopes that were recognized only by the natural human host (20) but did not react with sera from mice that were hyperimmunized to C. trachomatis (16). Subsequently, we comprehensively evaluated the utility of 11 top-ranked peptide antigens from 8 C. trachomatis proteins for use in human C. trachomatis serology (21). Results obtained for detection of antibodies against 4 commercial anti-C. trachomatis ELISA antigens and these 11 C. trachomatis peptide antigens showed that the peptide assays outperformed any of these commercial ELISAs both in specificity and sensitivity (21), a consequence of optimal peptide antigen design and ELISA protocol (16–21).

Importantly, the high sensitivity of the peptide assays was achieved mainly by the use of multiple B-cell epitopes of several C. trachomatis immunodominant proteins, including OmpA (21), compared to exclusive OmpA antigens used in commercial ELISAs. Given the stochasticity of antibody responses to individual B-cell epitopes (16, 20, 21), only the combined use of multiple peptide antigens can reliably measure host antibodies produced in response to C. trachomatis infection (21), similar to the quantitative results obtained with complex antigens. However, this requires single serum testing of individual peptide antigens in 11 separate microtiter wells (21). In addition, the optimally sensitive chemiluminescent ELISA format is not commonly used by the diagnostic laboratory community, which typically prefers colorimetric ELISA formats. Therefore, simplification of the current peptide ELISA methodology is urgently needed.

In the present study, we established a simple colorimetric ELISA using an antigen mixture of 12 strongly reactive C. trachomatis-specific peptide antigens (20), requiring per serum only single-well signal detection of anti-C. trachomatis antibodies. For test evaluation, we used sera from 125 women with NAAT-confirmed active C. trachomatis infection with high prevalence of anti-C. trachomatis antibodies (20, 21) and 87 negative-control sera. Importantly, this simple colorimetric ELISA outperformed all four commercial ELISAs for detection of anti-C. trachomatis IgG antibodies, with the advantage of higher sensitivity and specificity. Compared to the previously reported individual peptide assays (21), this simple mixed peptide ELISA achieved the same assay specificity with minimal loss of sensitivity but high labor savings.

## RESULTS

### Anti-C. trachomatis IgG antibodies in blood donor sera determined by commercial ELISAs.

**FIG 1:**
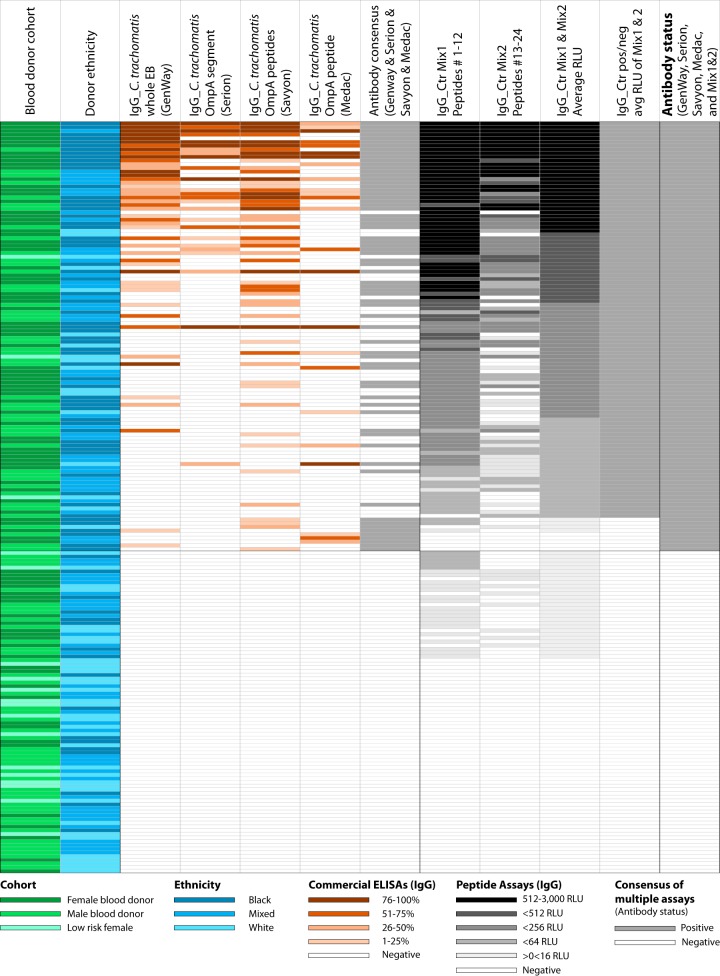
IgG reactivities of 203 blood donor sera with four commercial C. trachomatis ELISAs and two Ctr Mix1 and Ctr Mix2 peptide antigen mixtures. To identify a set of negative-control sera for anti-C. trachomatis antibodies, a panel of 203 sera was analyzed by 4 commercial colorimetric ELISAs and 2 highly sensitive chemiluminescent C. trachomatis*-*specific peptide antigen assays (21): (i) 18 healthy, low-risk women, (ii) 95 healthy women, and (iii) 90 healthy men. The first (leftmost) column indicates the origin of sera from either the cohorts of 95 female blood donors (dark green) or 90 male blood donors (medium green), or 18 low-risk women never diagnosed with C. trachomatis infection (light green). The next column shows the ethnic origin of the subjects: African American or black (dark blue), mixed race (medium blue), and Caucasian or white (light blue). The following 4 columns indicate percentile reactivity ranks of 4 commercial anti-C. trachomatis IgG ELISAs using the manufacturer-recommended cutoffs. Next, a consensus anti-C. trachomatis antibody status (positive/negative) is shown that assumed any serum as positive that was positive in any of the preceding 4 commercial C. trachomatis ELISAs. The next two columns show background corrected signals with Ctr Mix1 (peptides 1 to 12) and Ctr Mix 2 (peptides 13 to 24) for polyclonal antibody conjugate against human IgG, with reaction strength indicated by shading from white (nonreactive) to black (maximally reactive). The following column shows the average of relative light unit signals (RLU) for the Ctr Mix1 and Mix2 seroreactivities. The next column shows the binomial antibody status (positive or negative) based on average RLU signals of Ctr Mix1 and Mix2. The last column shows a final consensus of antibody status (positive/negative) based on the 4 commercial ELISAs and 2 peptide mix assays for anti-C. trachomatis IgG antibodies. The 87 consensus-negative sera were used as negative controls in the subsequent experiment (Fig. 2).

To identify a suitable cohort of negative-control sera for anti-C. trachomatis antibodies, we first tested 185 sera from 95 female and 90 male blood donors, as well as 18 sera from women at low risk of C. trachomatis infection, with four commercial ELISAs for detection of anti-C. trachomatis IgG. These ELISAs use either purified elementary bodies, an OmpA protein segment, or OmpA peptides. Anti-C. trachomatis IgG were detected in 25% (GenWay), 14% (Serion), 28% (Savyon), and 13% (Medac) of these sera, respectively, at manufacturer-defined ELISA cutoffs (Fig. 1). The cumulative results for the blood donor sera showed that 37% of the sera were positive for anti-C. trachomatis IgG (Fig. 1). In contrast, only 1 out of 18 women at low risk for C. trachomatis exposure (6%) (21) was positive for anti-C. trachomatis IgG (Fig. 1). This result suggests that a large portion of blood donors have been exposed to C. trachomatis and maintain high levels of anti-C. trachomatis IgG antibodies. Hence, to obtain a set of sera with low anti-C. trachomatis antibody prevalence, a substantial number of sera with high anti-C. trachomatis antibodies must be excluded from these blood donor sera.

### Anti-C. trachomatis IgG antibodies in blood donor sera determined by C. trachomatis-specific mixed peptide assay.

Next, the same set of sera was tested with a mixture of the 12 most reactive C. trachomatis peptide antigens (Ctr Mix1 [peptides 1 to 12] [Table 1]), as well as another mixture of 12 moderately reactive C. trachomatis peptide antigens (Ctr Mix2 [peptides 13 to 24]). To maximize sensitivity, chemiluminescent detection of bound polyclonal anti-human IgG HRP conjugate was used. At background plus 3×CV cutoff, 86% of female and 68% of male blood donors (140 out of 185) were positive for anti-C. trachomatis IgG antibodies. In contrast, only 28% (5 out of 18) sera from low-risk women were positive at this cutoff for anti-C. trachomatis IgG antibodies (Fig. 1).

**TABLE 1:** *Chlamydia trachomatis*-specific peptide antigens

Peptide antigen preparation	Peptide no.	Peptide antigen	Peptide sequence^*a*^	Human serum reactivity score^*b*^	Human host- dependent reactivity^*c*^	Sequence conservation (% identity)^*d*^	
						Ctr	Cpn
Ctr Mix1	1	CtrOmpA_313-339	IFDTTTLNPTIAGAGDVKTGAEGQLGD	10.3	No	74	<40
	2	CtrIncE_081-120	LFAISALDVLEDHGLVGCPFKLPCKSSPANEPTVQFFKGK	8.4	No	97	<40
	3	CtrPmpD_727-742	EKVEEVEPAPEQKDNN	8.2	Yes	100	<40
	4	CtrCT442_135-150	VVESLSRRNSLVDQTQ	8.9	Yes	99	<40
	5	CtrCT143_002-027	KKPVFTGGAPIPGISTEEGTGVKDQN	7.3	Yes	100	<40
	6	CtrCT529_200-239	SAERADCEARCARIAREESLLEVPGEENACEKKVAGEKAK	7.3	No	96	<40
	7	CtrTarP_116-145	TSSSDHIPSDYDDVGSNSGDISNNYDDVGS	6.3	Yes	77	<40
	8	CtrCT618_185-206	GNLKQNKPTEGTSKENGFMARL	7.4	Yes	99	<40
	9	CtrPmpD_1036-65	SGTPVQQGHAISKPEAEIESSSEPEGAHSL	6.8	No	98	<40
	10	CtrPmpD_536-565	ARAPQALPTQEEFPLFSKKEGRPLSSGYSG	7.7	No	100	<40
	11	CtrTarP_151-180	SSNYDDAAADYEPIRTTENIYESIGGSRTS	7.4	Yes	95	<40
	12	CtrCT813_235-264	AIENLDEMAYEAMEFEKEKHGIKPGRRGSI	7.2	Yes	97	<40

Ctr Mix2	13	CtrCT795_148-163	IMDITEIPSINPEFVE	7.0	Yes	99	<40
	14	CtrCT223_005-034	ALGTSNGVEANNGINDLSPAPEAKKTGSGL	7.0	Yes	97	<40
	15	CtrPmpC_483-498	APSLTEAESDQTDQTE	6.8	Yes	100	<40
	16	CtrPmpD_760-775	QALFASEDGDLSPESS	6.6	Yes	99	<40
	17	CtrIncE_117-132	FKGKNGSADKVILVTQ	6.5	Yes	97	<40
	18	CtrIncG_108-147	RPSDQQESGGRLSEESASPQASPTSSTFGLESALRSIGDS	6.5	No	98	<40
	19	CtrIncG_097-112	KRSPEEIEGAARPSDQ	6.4	Yes	100	<40
	20	CtrPmpC_608-637	AIVESTPEAPEEIPPVEGEESTATEDPNSN	6.4	Yes	99	<40
	21	CtrCT813_203-232	TVTDLEAAKQQLEEKVTDLESEKQELREEL	6.0	Yes	100	<40
	22	CtrCT875_398-427	KGSTHRYAPRDDLSPEGASLAETLARFADD	5.9	Yes	100	<40
	23	CtrCT579_256-286	ALDDVAGTATAVGAKATSGAASAASSAATK	5.6	Yes	100	42
	24	CtrLcrE_392-421	RSSFSSTPPHAPVPQSEIPTSPTSTQPPSP	5.4	Yes	99	<40

a Only the actual *C. trachomatis* serovar D strain D/UW-3/Cx peptide antigen sequences are shown, without N-terminal biotin and serine-glycine-serine-glycine spacer that is attached to each peptide (16).

b Reactivity scores are log_2_ weighted averages of seven reactivities of total and subpooled sera as described previously (20).

c Reactivity with only human *C. trachomatis*-specific immune sera (yes) or also with mouse anti-*C. trachomatis* hyperimmune mouse sera (no) is shown (16, 20).

d Average percent amino acid sequence identity with 22 *C. trachomatis* strains representing all major *C. trachomatis* clades or 5 *C. pneumoniae* strains representing all major *C. pneumoniae* clades is shown (20). Ctr, *C. trachomatis*; Cpn, *C. pneumoniae*.

### Determination of host exposure to C. trachomatis by colorimetric ELISA with mixed C. trachomatis peptide antigens.

**FIG 2:**
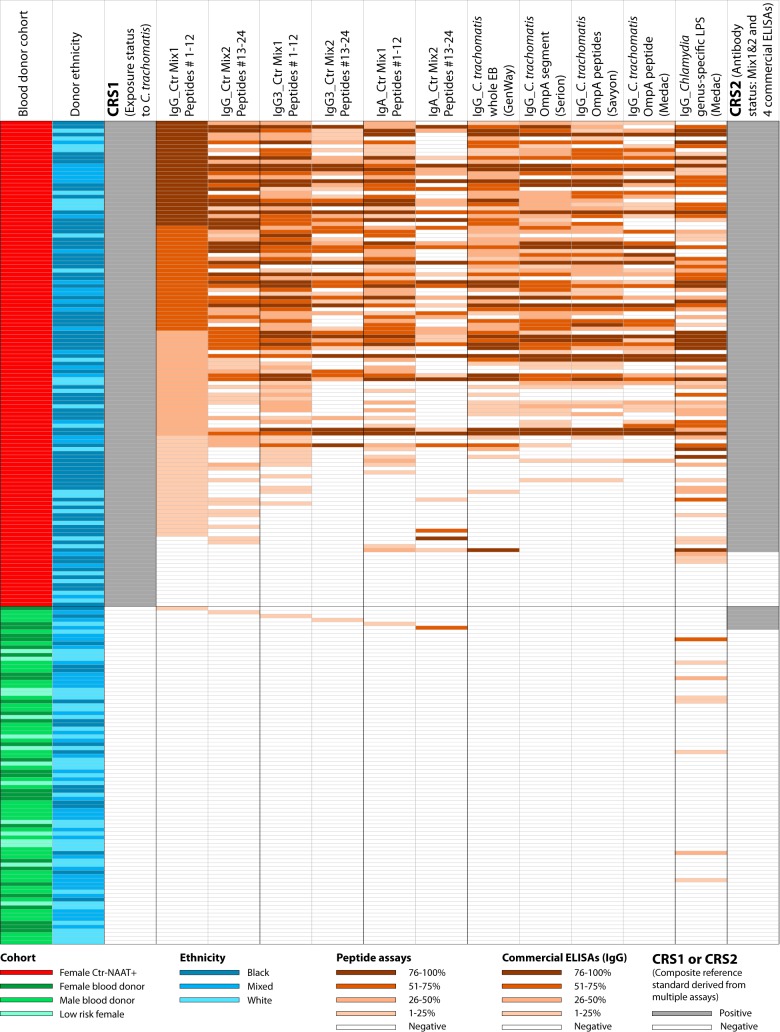
Detection sensitivity of anti-C. trachomatis antibodies in sera from Ctr-NAAT-positive women. An artificial population was created by combining 125 high anti-C. trachomatis antibody-prevalence sera from Ctr-NAAT-positive women (21) and 87 sera that had tested negative in multiple previous Ctr antibody assays (Fig. 1). The first (leftmost) column indicates origin of sera from either the cohort of 125 Ctr NAAT-positive women (red) or the 3 cohorts of low Ctr-antibody-prevalence sera shown in Fig. 1. The next column shows the ethnicity of the subjects as in Fig. 1, followed by the composite reference standard CRS1 for NAAT-proven exposure to C. trachomatis (Table 2). The next columns show background-corrected colorimetric signals of Ctr Mix1 and Ctr Mix 2 for an anti-human IgG polyclonal antibody conjugate, as well as monoclonal antibody conjugates for detection of short-lived IgG3 and IgA1&A2 antibody isotypes. The cutoff in these assays was chosen at 98.9% or higher specificity, with negative (white) and percentile reactivity ranking from low (light brown) to maximal (dark brown). The next columns indicate reactivity in 4 anti-C. trachomatis IgG commercial ELISAs as partially shown in Fig. 1, followed by a *Chlamydia* genus-specific ELISA for detection of IgG antibodies against chlamydial LPS. The last column shows a consensus antibody status (positive/negative) based on both 4 commercial ELISAs and 2 peptide assays for anti-C. trachomatis IgG, IgG3, and IgA antibodies. This consensus status, CRS2, was used for ROC analyses that evaluated the performance of the individual assays (Table 3).

For initial evaluation of colorimetric ELISAs for detection of anti-C. trachomatis antibodies, a composite reference standard 1 (CRS1) for active C. trachomatis infection status was constructed using 125 exposure-positive and 87 exposure-negative sera (Fig. 2). Sera from 125 C. trachomatis NAAT-positive women served as C. trachomatis exposure-positive sera in CRS1 (Fig. 2). From the cohort of 185 blood donors and 18 low-risk women, a set of 87 sera with no or minimally detectable anti-C. trachomatis IgG antibodies (Fig. 1) was chosen as negative-control sera for CRS1 (Fig. 2). These sera were negative in the four commercial ELISAs for anti-C. trachomatis IgG at manufacturer-defined cutoffs, as well as negative at 16 RLU cutoff in the two chemiluminescence Ctr Mix1 and Mix2 ELISAs (Fig. 1).

We then established a simple colorimetric ELISA for single-signal detection of anti-C. trachomatis antibodies with the Ctr Mix1 and Mix2 peptide antigens. Using CRS1, we evaluated the sensitivity of the colorimetric Ctr Mix1 and Mix2 ELISAs for detection of C. trachomatis infection (Fig. 2 and Table 2). At ≥98.9% specificity for the negative sera, the Ctr Mix1 had approximately 86%, 70%, and 60% sensitivity for detection of anti-C. trachomatis IgG, IgG3, and IgA antibodies, respectively, among the NAAT+ sera. Ctr Mix2 at ≥98.9% specificity achieved 63%, 42%, and 30% sensitivity, respectively, substantially lower than Ctr Mix1 (Fig. 2). The 86% and 63% sensitivities of Ctr Mix1 and Mix2 peptides for anti-C. trachomatis IgG compare favorably to those of commercial anti-C. trachomatis IgG ELISAs with 61% (GenWay), 58% (Serion), 53% (Savyon), and 42% (Medac) when tested with the same population of NAAT+ sera (21). Importantly, compared to commercial ELISAs, the single-signal detection with Ctr Mix1 achieved 25% to 44% higher sensitivity (Table 2). Thus, despite an active C. trachomatis infection in the NAAT+ population, a large proportion of these sera (39%) remained negative in the best commercial ELISA. In contrast, a much smaller proportion (14%) of these sera remained negative in the single-well Ctr Mix1 IgG ELISA, resulting in 41% improved assay sensitivity over the commercial GenWay ELISA (*P < *10^−5^, Fisher exact test).

**TABLE 2:** Assay sensitivity for detection of host exposure to *C. trachomatis*^*a*^

Detected antibody isotype	Antigen	Sensitivity (%)^*b*^
IgG	Ctr Mix1^*c*^	85.6
	Ctr Mix2^*c*^	63.2

IgG3	Ctr Mix1	70.4
	Ctr Mix2	41.6

IgA (IgA1&IgA2)^*d*^	Ctr Mix1	60.0
	Ctr Mix2	30.4

IgG	Ctr EB (GenWay)	60.8
	Ctr OmpA (Serion)	57.6
	Ctr OmpA_Pept (Savyon)	52.8
	Ctr OmpA_Pept (Medac)	41.6

IgG	*Chlamydia* rLPS (Medac)	60.8

a Assay sensitivities were determined using CRS1 constructed from data for 212 sera (Fig. 2). Exposure to *C. trachomatis* was determined by NAAT positivity and nonexposure by anti-*C. trachomatis* antibody negativity in multiassay testing (Fig. 1).

b For 4 commercial anti-*C. trachomatis* IgG ELISAs, sensitivity was calculated using manufacturer-defined cutoffs. For Ctr Mix1 and Mix2 peptide assays, a cutoff was chosen that classified 1 out of 87 sera as falsely positive (98.9% specificity).

c Ctr Mix1 and Ctr Mix2 indicate mixtures of 12 peptides (Table 1) that were tested in a single microtiter well.

d The ampersand indicates that a mixture of two monoclonal anti-human IgA1 and IgA2 conjugates was used for a single IgA antibody signal readout.

### Anti-C. trachomatis antibodies determined by colorimetric ELISAs in the simulated test population of pooled low and high anti-C. trachomatis antibody prevalence cohorts.

For determination of anti-C. trachomatis antibody status, a second composite reference standard (CRS2) was established for the simulated test population of pooled low and high anti-C. trachomatis antibody prevalence cohorts (Fig. 2). For CRS2, any serum was considered positive if it was positive in any one of the assays shown in Fig. 2, resulting in 117 sera as positive and 95 sera as negative for anti-C. trachomatis antibodies. A small fraction of NAAT-positive individuals (11.2%) were negative for anti-C. trachomatis antibodies in all tests (Fig. 2). This incongruent outcome may be caused by the true absence of an antibody response against C. trachomatis in certain individuals and/or low sensitivity of the antibody detection assays. Using CRS2 as the gold standard for anti-C. trachomatis antibody status, the sensitivities of individual assays at different specificity cutoffs were calculated from ROC curves (Table 3). At 98% specificity, the Ctr Mix1 antigens (peptides 1 to 12) achieved 93.9% sensitivity for anti-C. trachomatis IgG antibodies and 81.6% sensitivity for short-lived IgG3+IgA antibodies (Table 3). In contrast, the sensitivities of commercial IgG ELISAs at the same 98% specificity were 78.7% for GenWay, 70.2% for Serion, 48.8% for Savyon, and 54.9% for Medac (Table 3). Thus, even at high specificity (98%), the Ctr Mix1 showed substantially higher sensitivity (15.2% to 45.1%) than four commercial ELISAs (*P < *10^−5^, Fisher exact test). Compared to the performance achieved by Ctr Mix1, the Ctr Mix2 peptide antigens performed moderately with a 69.4% sensitivity for IgG.

**TABLE 3:** Anti-*C. trachomatis* antibody assay sensitivities at different assay specificities^*a*^

Antigen	Detected antibody isotype^*b*^	Sensitivity (%)					Avg^*c*^ sensitivity at specificity of 98 to 80%	AUC
		Sp of 98%	Sp of 95%	Sp of 90%	Sp of 85%	Sp of 80%		
*Chlamydia* rLPS (Medac)	IgG	38.5	53.4	66.3	74.1	79.6	**62.4**	0.881

Ctr EB (GenWay)	IgG	78.7	83.1	86.5	88.5	89.9	**85.3**	0.933
Ctr OmpA (Serion)	IgG	70.2	75.2	79.3	81.8	83.6	**78.0**	0.887
Ctr OmpA_Pept (Savyon)	IgG	48.8	58.3	66.4	71.4	75.2	**64.0**	0.844
Ctr OmpA_Pept (Medac)	IgG	54.9	68.1	78.3	83.9	87.7	**74.6**	0.923

Ctr Mix1	IgG	93.9	94.7	95.4	95.9	96.2	**95.2**	0.971
	IgG3	75.5	76.8	79.4	80.2	81.5	**78.7**	0.883
	IgA (IgA1&IgA2)	66.4	73.1	78.4	81.6	83.9	**76.7**	0.895
	IgG3+IgA	81.6	84.7	87.1	88.6	89.7	**86.3**	0.926

Ctr Mix2	IgG	69.4	75.1	79.6	82.4	84.4	**78.2**	0.895
	IgG3	45.4	47.6	50.7	53.2	56.1	**50.6**	0.727
	IgA (IgA1&IgA2)	37.0	45.8	53.9	59.2	63.2	**51.9**	0.760
	IgG3+IgA	46.3	54.3	61.3	65.8	69.3	**59.4**	0.795

a Assay sensitivities were determined by ROC curves of each individual assay that were constructed from the CRS2 data of 117 anti-*C. trachomatis* antibody-positive and 95 antibody-negative sera (Fig. 2). The binomial anti-*C. trachomatis* antibody status was used as *X* categorical variable known *a priori*, and serum seroreactivities were used as *Y* continuous predictor variable. The anti-*C. trachomatis* antibody status of these sera was determined from the antibody-positive/negative results in four commercial IgG ELISAs and in IgG, IgG3, and IgA reactivities of Ctr Mix1 and Ctr Mix2 peptide antigens (Fig. 2). Sp, specificity.

b The plus sign indicates that two conjugates were used in two separate reactions resulting in two signal readouts, and the average of these two signals was used in the analysis.

c The average sensitivity was calculated from the sensitivities at five different assay specificities (98%, 95%, 90%, 85% and 80%).

At less stringent cutoff values such as 95% to 80% specificity, the sensitivities of all assays increased; this was most pronounced for the commercial IgG ELISAs (Table 3). Nonetheless, at 5 different assay cutoffs from 98% to 80% specificities, the Ctr Mix1 peptide antigens (peptides 1 to 12) achieved an average sensitivity of 95.2% for anti-C. trachomatis IgG detection, 10% to 30% higher than the average sensitivity of 64% to 85.3% of the commercial IgG ELISAs (*P < *0.01, paired Student’s *t* test). These results, in addition to our previous reports (21), clearly establish that mixed multipeptide assays substantially outperform available commercial ELISAs. Unlike Ctr Mix1, the less immunodominant C. trachomatis B-cell epitopes in Ctr Mix2 (peptides 13 to 24) did not achieve high assay sensitivity. Thus, the high performance of Mix1 stems from the use of strongly reactive peptide antigens derived from several immunodominant proteins.

Another commercial IgG ELISA based on recombinant *Chlamydia* genus-specific lipopolysaccharide (LPS) was tested because of its common use. It performed poorly compared to the consensus anti-C. trachomatis antibody status (Table 3) and showed 68.5% sensitivity for detection of anti-C. trachomatis IgG antibodies at the manufacturer-defined cutoff (Fig. 2). In addition, the LPS antigen of this Medac ELISA also detected antibodies in 11.6% of anti-C. trachomatis antibody-negative sera (Fig. 2), probably by cross-reactivity with anti-C. pneumoniae antibodies. Thus, because of poor sensitivity and cross-reactivity with antibodies against LPS from other *Chlamydia* spp., this Medac rLPS ELISA is not suitable for specific and/or sensitive detection of anti-C. trachomatis antibodies (Fig. 2).

### Diagnostic suitability of assays determined by likelihood ratios.

**FIG 3:**
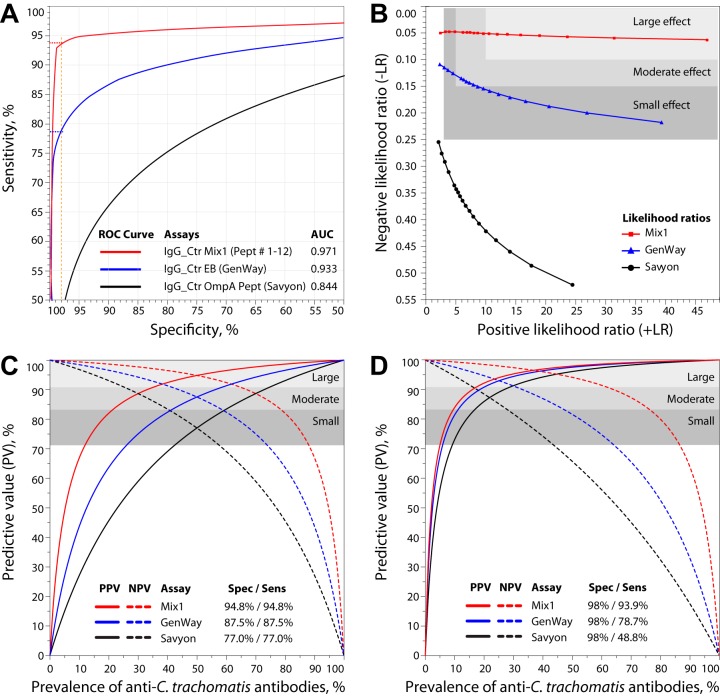
Diagnostic utility modeling of anti-C. trachomatis antibody assays by ROC curves, likelihood ratios, and predictive values. The antibody consensus of 125 C. trachomatis infection-positive sera and 87 Ctr antibody-negative sera (Fig. 2 and Table 2) was used as categorical variable known *a priori*, and the observed serum reactivities in a test were used as predictor variables of the anti-C. trachomatis antibody status. (A) ROC evaluation of individual anti-C. trachomatis antibody assays against the consensus antibody status. Solid color lines indicate maximum likelihood-fitted ROC curves. The dotted lines indicate sensitivity at 98% specificity. (B) Positive (+LR) and negative (−LR) likelihood ratios are independent of antibody prevalence. Sensitivities were calculated from ROC curves at specificities ranging from 60% (left) to 98% (right). The best performance of an assay is found at high +LR and low −LR (top right). The three gray shaded areas indicate the zones of high, moderate, and poor performance of the assays, corresponding to large, moderate, and small diagnostic effects (both +LR and −LR). (C and D) Positive and negative predictive values in dependence of population prevalence of anti-C. trachomatis antibodies were modeled for high (C) and low (D) prevalence. Predictive values for anti-C. trachomatis antibody status were calculated using ROC-derived specificity and resultant sensitivity.

Next, we sought to comparatively assess the diagnostic utility of the colorimetric Ctr Mix1 peptide IgG assay and the commercial anti-C. trachomatis IgG ELISAs by use of likelihood ratios. For the Ctr Mix1 IgG assay (Table 3), any 91% to 98% specificity cutoff corresponding to ∼94% sensitivities (Fig. 3A) achieved large diagnostic effect sizes for both positive likelihood ratios (+LR = 10.6 to 46.9) and negative likelihood ratios (−LR = 0.05 to 0.06) (Fig. 3B). In contrast, the top performing commercial GenWay ELISA (Table 2) achieved only moderate diagnostic effect sizes for both +LR (5.9 to 8.6) and −LR (0.14 to 0.15) (Fig. 3B) at 85% to 90% specificity cutoffs with corresponding ∼88.5% to 86.5% sensitivities (Fig. 3A). At +LR from 5 to 25 (Fig. 3B), the 0.05 −LR Ctr Mix1 peptide reactivity is highly significantly better than the 0.16 −LR of the GenWay ELISA (*P* =10^−4^, paired Student’s *t* test).

### Diagnostic suitability of assays determined by predictive value modeling in dependence on anti-C. trachomatis prevalence.

Finally, we used predictive value modeling to comparatively assess the diagnostic utility of the colorimetric Ctr Mix1 peptide IgG assay and the commercial anti-C. trachomatis IgG ELISAs in hypothetical populations with 0% to 100% prevalence of anti-C. trachomatis antibodies. For 50% antibody prevalence, if equal assay sensitivity and specificity are chosen from ROC curves, the positive predictive value (PPV) and negative predictive value (NPV) become equal (Fig. 3C). Quantitative comparison at 50% prevalence shows substantially higher PPV and NPV for the Ctr Mix1 assay than for commercial ELISAs (Fig. 3C). At 94.8% specificity and sensitivity, the Ctr Mix1 IgG assay achieved high (PPV&NPV ≥ 90.9%) and moderate (PPV&NPV ≥ 83.3%) performance for 36% to 64% and 22% to 78% prevalence, respectively (Fig. 3C). For the top performing GenWay ELISA, a significantly smaller (*P* <0.005) diagnostic effect size (poor performance, PPV&NPV ≥ 71.4%) was obtained for 27% to 73% prevalence (Fig. 3C).

For low prevalence populations, high specificity cutoffs are required, and uniformly 98% assay specificity was chosen for all assays for comparative performance evaluation (Fig. 3D). This resulted in a sensitivity of 93.9% for the Ctr Mix1 IgG assay, but insufficient sensitivities of 78.7% and 48.8% for the GenWay and Savyon ELISAs, respectively. The C. trachomatis peptide IgG assay achieved high and moderate performance for prevalence ranges from 18% to 61% and 10% to 76%, respectively. In contrast, the commercial ELISAs at best achieved a significantly reduced (*P* < 0.01) moderate performance at 12% to 47% (GenWay) and 17% to 27% prevalence (Savyon) (Fig. 3D). It is important to note that the low sensitivity of the commercial ELISAs results in a precipitous NPV drop if a high specificity cutoff (and PPV) is chosen.

### Concordance of Ctr Mix1 with commercial and Ctr Mix2 ELISAs.

**FIG 4:**
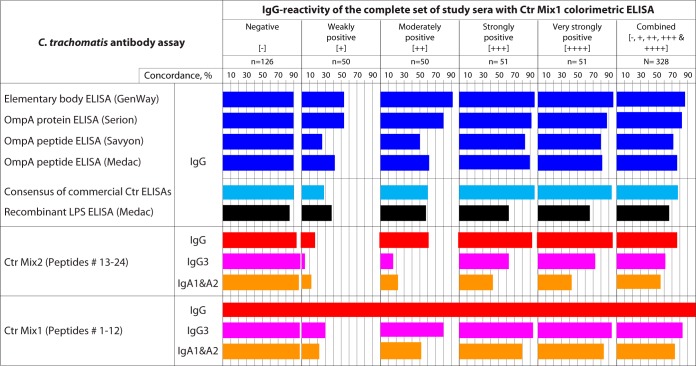
Concordance of Ctr Mix1 reactivity with commercial and Ctr Mix2 ELISAs. Out of 328 total study sera, the 202 peptide-reactive sera were scored +1 to +4 based on their reactivity rank. The cutoff of Ctr Mix1 ELISA was chosen at 98.9% specificity (Fig. 2), and cutoffs for the remaining assays were readjusted to maximize concordance with the Ctr Mix1 ELISA.

At 98.9% specificity cutoff for Ctr Mix1 ELISA (CRS2 in Fig. 2), 202 sera among the complete set of 328 study sera were classified as positive and 126 as negative for anti-C. trachomatis antibodies (Fig. 4). For the 202 Ctr-positive sera, quartile Ctr Mix1 IgG reactivity rank scores of +1 to +4 (Fig. 4) were assigned to 50, 50, 51, and 51 sera, respectively. The four commercial ELISAs showed high concordance (80% to 96%) for strongly reactive Ctr Mix1-positive sera as well for Ctr Mix1-negative sera (∼90%). In contrast, these commercial ELISAs showed poor concordance (26% to 54%) for weakly reactive Ctr Mix1-positive sera (Fig. 4). The Ctr Mix2 ELISA showed 92% to 95% concordance for very strongly reactive, strongly reactive, or nonreactive Ctr Mix1 IgG sera, but only 18% concordance with weakly reactive Ctr Mix1 IgG-positive sera. The low concordance of sera with low levels of anti-C. trachomatis IgG indicates poor assay sensitivities of the commercial and Ctr Mix2 ELISAs. Overall, the Ctr Mix1 seroreactivities were 87%, 83%, 72%, and 77% concordant with the GenWay, Serion, Savyon, and Medac ELISAs, respectively. Compared to IgG isotype reactivities in the Ctr Mix1 ELISA, the reactivities of short-lived IgG3 and IgA1&A2 antibody isotypes showed high concordance for strongly reactive sera but poor concordance for weakly reactive sera. As expected, these results indicate that high IgG titer is also associated with frequent detection of short-lived IgG3 and IgA1&IgA2 antibody isotypes.

### Prevalence of anti-C. trachomatis antibodies in the sera from 4 cohorts.

Among the cohorts of 125 women with NAAT-proven active C. trachomatis infections, 95 female blood donors, 90 male blood donors, and 18 low Ctr-infection risk females (Table 4), the consensus of four commercial IgG ELISAs identified anti-C. trachomatis IgG antibodies in 68%, 39%, 34%, and 6% of each cohort, respectively. In contrast, the Ctr Mix1 detected anti-C. trachomatis IgG antibodies in 86%, 63%, 38%, and 11% of the sera from these cohorts (Table 4). Thus, compared to the commercial ELISAs, the novel colorimetric Ctr Mix1 peptide ELISA showed substantially higher assay sensitivity (18% to 24%) for anti-C. trachomatis antibody detection in women (*P < *0.001, chi-square test). This effect was most pronounced in Caucasian female blood donors, with a 3.3-fold increased detection frequency (*P = *0.041, two-tailed Fisher exact test). In addition, the Ctr Mix1 is very well suited for detection of short-lived IgA+IgG3 antibody isotypes, with detection frequencies of 60% to 70%, 34% to 40%, 18% to 29%, and 6% to 11% in the sera from these four cohorts (Table 4). Finally, the Ctr Mix1 confirms the well-known tendency of higher anti-C. trachomatis antibody frequencies in women versus men (*P < *0.001, two-tailed Fisher exact test), as well as in African American individuals versus in individuals of Caucasian or other origin (*P < *10^−4^, two-tailed Fisher exact test).

**TABLE 4:** Anti-*C. trachomatis* antibody-positive sera of all cohorts

Cohort (*n*) and ethnicity (*n*)	Commercial ELISAs (% pos)^*a*^		Ctr Mix1 peptide reactivity (% pos)^*b*^							
	IgG		IgG		IgG3		IgA		IgG3+IgA	
**NAAT+, female (125)**										
African American (74)	66.2		87.8		70.2		62.2		74.3	
Caucasian (28)	60.7	**68.0**	78.6	**85.6**	57.1	**70.4**	50.0	**60.0**	71.4	**76.0**
Other (23)	82.6		87.0		87.0		65.2		87.0	

**Blood donor, female (95)**										
African American (38)	63.2		81.6		65.8		50.0		73.7	
Caucasian (20)	15.0	**38.9**	50.0	**63.2**	10.0	**40.0**	15.0	**33.7**	20.0	**47.4**
Other (37)	27.0		51.4		29.7		27.0		35.1	

**Blood donor, male (90)**										
African American (39)	48.7		48.7		38.5		20.5		43.6	
Caucasian (15)	13.3	**34.4**	20.0	**36.7**	6.7	**28.9**	20.0	**17.8**	20.0	**33.3**
Other (36)	27.8		30.6		27.8		13.9		27.8	

**Low Ctr risk, female (18)**										
Caucasian (18)	5.6	**5.6**	11.1	**11.1**	11.1	**11.1**	5.6	**5.6**	11.1	**11.1**

a For four commercial *C. trachomatis* IgG ELISAs, reactivity cutoffs defined by the manufacturers were used, and a serum was scored positive if any one of the ELISAs was positive. The values are the percentage positive. Boldface values are the average for the cohort.

b For each antibody isotype-specific peptide reactivity, a cutoff was chosen so that only 1 out of the 87 anti-*C. trachomatis* antibody-negative sera in Fig. 2 was falsely classified as positive (98.9% specificity cutoff). Boldface numbers are the average for the cohort.

### Comparative assay sensitivities of mixed and individual C. trachomatis peptide antigen ELISAs.

**FIG 5:**
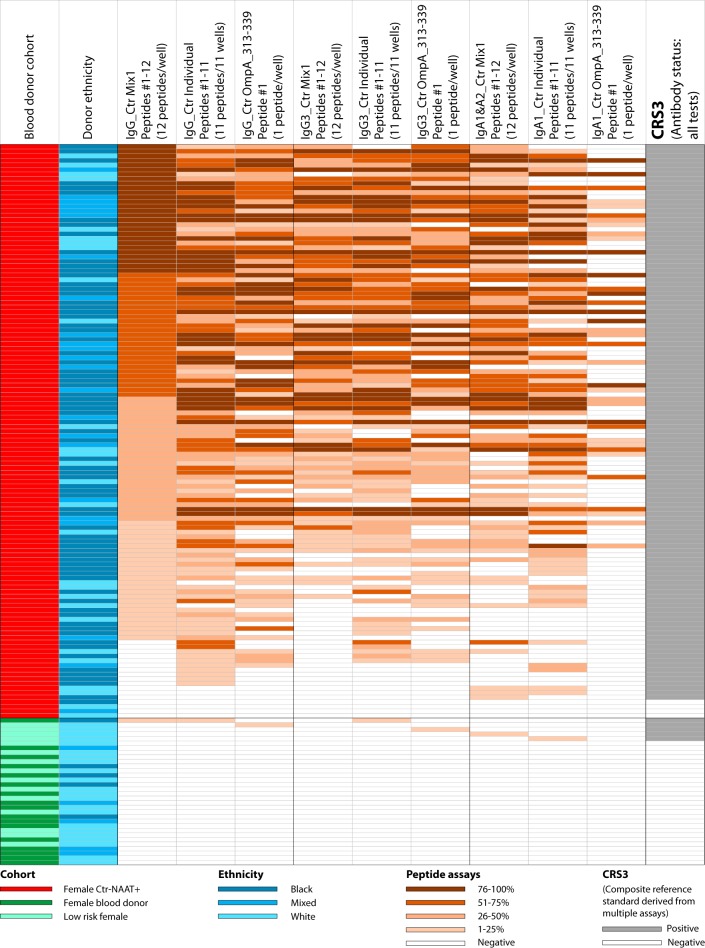
Comparative assay sensitivities of mixed and individual C. trachomatis peptide antigen ELISAs. The performance of the best Ctr Mix1 mixed peptide assay (12 peptides per microtiter well) was compared to the anti-C. trachomatis antibody status that had been previously determined by combining individual test results of 11 of these peptides, or by the single test result of the top-ranked OmpA peptide (21). The sera used were from 125 Ctr NAAT-positive women (Fig. 2) and from 32 anti-C. trachomatis antibody-negative blood donors or low-risk women. The columns indicate first (leftmost) the cohort and ethnic origin of the sera, followed by background-corrected anti-IgG OD signals of Ctr Mix1 (peptides 1 to 12), the average RLU reactivity of 11 individually tested peptides (peptides 1 to 11), and the single RLU reactivity of the CtrOmpA_313-339 peptide. Similarly, the next columns show seroreactivities for the short-lived human IgG3 antibody isotype, followed by the IgA isotype. The last column shows a consensus of anti-C. trachomatis antibody status (positive/negative) based on all peptide assays. This consensus status, CRS3, was used for ROC performance analyses of individual assays (Table 4).

In previous studies, we had tested the 125 sera of all NAAT+ women and 32 anti-C. trachomatis antibody-negative sera with 11 individual peptide antigens (21). Here, in Fig. 5 we compare the individual peptide assays of these 157 sera with the single Ctr Mix1 assay, using CRS3 of 126 positive and 31 negative sera as the gold standard for anti-C. trachomatis antibody detection. The sensitivities of individual assays at different specificity cutoffs were calculated from ROC curves (Table 5). At 98% to 80% specificity cutoff, the Ctr Mix1 (peptides 1 to 12) achieved an average 89.9% Ctr-IgG sensitivity and 80.4% Ctr-(IgG3+IgA) sensitivity (Table 5). In contrast, at the same specificity, the sensitivities of the combined average reactivity of the individual 11 C. trachomatis peptide antigens (peptides 1 to 11) were 95.1% for IgG (IgG1+IgG3) and 93.3% for IgG3+IgA1 (Table 5). In addition, at 98% specificity, the single CtrOmpA_313-339 peptide achieved 82.3% sensitivity for IgG and 76.0% for IgG3+IgA1 detection (Table 5). Importantly, the 70.8% to 80.7% sensitivity of the four commercial ELISAs was lower than the 82.3% IgG assay sensitivity of the single OmpA peptide (Table 5).

**TABLE 5:** Determination of anti-*C. trachomatis* antibody status by mixed versus individual peptide assay^*a*^

Antigen	Antibody	Sensitivity (%)					Avg sensitivity at specificity of 98% to 80%	AUC
		Sp of 98%	Sp of 95%	Sp of 90%	Sp of 85%	Sp of 80%		
Ctr Mix1 (peptides 1 to 12)	IgG	86.2	88.6	90.5	91.6	92.5	**89.9**	0.947
	IgG3	70.5	71.5	73.5	75.5	77.0	**73.6**	0.853
	IgA (IgA1&IgA2)	70.0	73.1	74.3	74.5	75.0	**73.4**	0.816
	IgG3+IgA	79.0	80.1	80.3	81.0	81.5	**80.4**	0.866

Avg of individual Ctr peptide 1 to 11 reactivities	IgG (IgG1+IgG3)	92.4	94.3	95.6	96.4	96.9	**95.1**	0.980
	IgG3	82.0	88.1	92.1	94.2	95.5	**90.4**	0.970
	IgA1	69.5	70.0	71.9	73.5	75.5	**72.1**	0.845
	IgG3+IgA1	83.9	91.5	95.6	97.4	98.3	**93.3**	0.983

CtrOmpA_313-339	IgG (IgG1+IgG3)	80.1	81.2	82.5	83.0	84.5	**82.3**	0.901
	IgG3	70.2	71.3	72.9	74.5	76.8	**73.1**	0.850
	IgA1	29.9	01.5	36.5	39.7	42.6	**30.0**	0.643
	IgG3+IgA1	72.5	73.0	75.1	76.4	87.9	**76.0**	0.861

*Chlamydia* rLPS (Medac)	IgG	35.1	51.3	65.6	74.3	80.2	**61.3**	0.884

Ctr EB (GenWay)	IgG	70.1	77.1	82.5	85.7	87.9	**80.7**	0.923
Ctr OmpA (Serion)	IgG	59.8	66.8	72.6	76.2	78.8	**70.8**	0.859
Ctr OmpA_Pept (Savyon)	IgG	62.6	72.0	79.2	83.3	86.2	**76.7**	0.915
Ctr OmpA_Pept (Medac)	IgG	64.1	75.1	83.1	87.5	90.3	**80.0**	0.939

a Assay sensitivities were determined by ROC curves of each individual assay that were constructed from the CRS3 data of 126 anti-*C. trachomatis* antibody-positive and 31 antibody-negative sera (Fig. 4). Anti-*C. trachomatis* antibody status in CRS3 was determined by combining reactivities with the Ctr Mix1 peptides, the average of 11 individually tested peptides, and the single OmpA peptide antigen (Fig. 4).

Collectively, compared to 11 individual peptide reactivity assays, the single colorimetric signal detection of anti-C. trachomatis antibodies by the Ctr Mix1 peptide antigens showed 5.2% lower assay sensitivity for IgG and 12.9% lower sensitivity for IgG3+IgA (Table 5). However, compared to the best performing single OmpA peptide antigen, the Ctr Mix1 achieved 7.6% and 4.4% higher sensitivity for IgG and IgG3+IgA detection, respectively. Importantly, compared to the commercial IgG ELISAs, the Ctr Mix1 sensitivity achieved substantially higher sensitivity (9.2% to 19.1%). Thus, the single-well Ctr Mix1 ELISA outperformed all other tests except for the labor-intensive combined average of 11 individually tested best peptide antigens (Table 5).

### Relative ELISA signal amplitude of peptide antigens tested individually versus in mixture.

**FIG 6:**
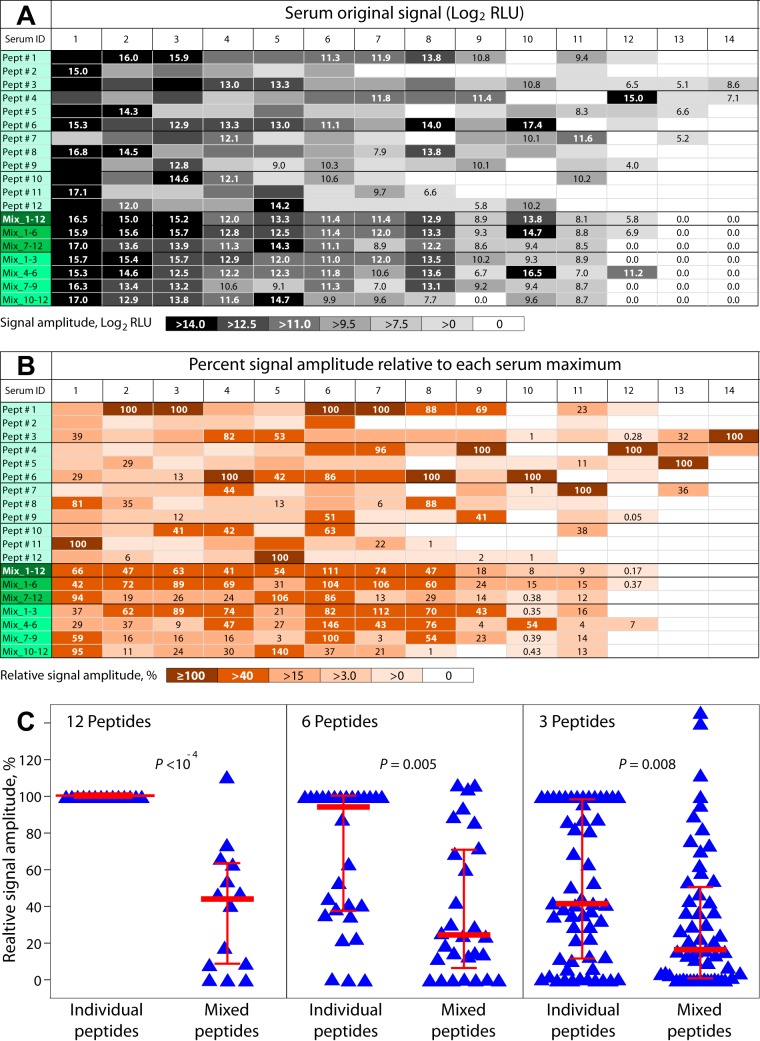
Original and relative signal amplitude of peptides tested singly or in mixtures with 14 reactive sera. Fourteen sera representing a high to low reactivity spectrum (left to right) were tested in chemiluminescent ELISA format to compare the signals of 12 individually tested peptide antigens to signals produced by peptide mixtures containing these antigens. Microtiter wells were coated with a total of 25 pM peptides, either as single peptides or as equimolar mixes of 12, 6, or 3 peptides. This represents an approximately 10 to 20× amount of biotinylated peptides required to saturate the streptavidin binding sites for biotin. (A) The original signals in log_2_ RLU of single peptides and their mixtures are shown in columns for each serum. Twelve individual immunodominant C. trachomatis peptide antigens (peptides 1 to 12) and seven antigen mixtures of peptides 1 to 12 (all 12 peptides), peptides 1 to 6, peptides 7 to 12 (2 × 6 peptides), peptides 1 to 3, peptides 4 to 6, peptides 7 to 9, or peptides 10 to 12 (4 × 3 peptides) were tested. Signal amplitude is shown by the color intensity of each field, and the numbers indicate the signal of the strongest reactive individual peptide among each set of three peptides or the signal of one of the mixtures indicated below individual peptides. (B) The maximum signal among the 12 individual peptides with each serum was considered 100%, and all other signals of individual and mixed peptides were transformed to a percentage relative to each serum maximum. (C) Comparison of relative signal amplitude of peptides tested individually and in mixtures of 12, 6, or 3 peptides. The maximal signal among the 12 individual peptides with each serum was considered 100%. Reactivities of individual and mixed peptides were plotted relative to each serum maximum, and medians ± 25 to 75 percentiles are shown. For the 12-peptide mix, the RLU signal of a specific serum with the complete Ctr Mix1 (peptides 1 to 12) was compared to the maximal individual signal among the 12 constituent Ctr Mix1 peptide antigens. For the 6-peptide mixes, the serum-specific signals with two Ctr Mix1 subsets (peptides 1 to 6 and 7 to 12) were compared to the serum maxima. For the 3-peptide mixes, the serum-specific signals with four Ctr Mix1 subsets (peptides 1 to 3, peptides 4 to 6, peptides 7 to 9, and peptides 10 to 12) were compared to the serum maxima.

To compare ELISA signal amplitudes produced by 12 individual peptides and their mixtures of all 12 peptides, 2 × 6, or 4 × 3 peptides, 14 individual sera with high to low reactivity spectrum were tested in chemiluminescent ELISA format with these 12 individual peptide antigens coated on 12 separate microtiter wells as well as each peptide mixture in a separate well as shown in Fig. 6. All 14 sera were reactive with 2 to 12 individual peptide antigens (Fig. 6A). Under the same assay condition, sera 13 and 14 were completely nonreactive with all 7 mixtures of 12, 6, or 3 peptide antigens, and serum 12 was only marginally reactive with 3 peptide antigen mixtures (Fig. 6A and B). Importantly, relative signals obtained with peptide mixtures were typically lower than the maximum signal among the 12 individual peptides (*P *≤* *0.008, Kruskal-Wallis test) (Fig. 6C). These data indicate unequivocally that the overwhelming majority of peptide reactivities in mixed peptide assays are not additive but rather reduced compared to the reactivity of individually tested peptides.

## DISCUSSION

In this study, we established a simple colorimetric ELISA for detection of anti-C. trachomatis antibodies by a single signal from multiple mixed peptide antigens. We used a mixture of 12 strongly reactive peptide antigens (Ctr Mix1 [Table 1]) that had previously been identified by use of hyperimmune mouse antisera against C. trachomatis (16, 20) and human sera from women with active C. trachomatis infection (20, 21). Mixing of these peptide antigens in a single reaction achieves the same high specificity with only a marginal loss of sensitivity (Table 5) compared to labor-intensive individual testing of each of the same peptides (21). Actual costs would be similar to traditional colorimetric ELISA, except for the one-time costs of peptide synthesis (<$2,000 for 12 peptides of 16 to 40 amino acids [aa]). Since these peptide antigens can be used for thousands of microtiter plates, the costs per assay would depend on the number of microtiter plates produced, the consumables per plate, and labor. Thus, we anticipate that such mixed peptide ELISAs will be highly labor-saving, economical, highly specific with adequate sensitivity, and suitable when small-volume samples are available such as sera eluted from dried blood spots (22, 23).

These highly immunodominant C. trachomatis-specific B-cell epitope peptide antigens capture host antibodies against a wide range of C. trachomatis proteins (OmpA, IncE, PmpD, CT442, CT143, CT529, TarP, CT618, and CT813 [Table 1]). Our and others’ previous studies showed that use of complex antigens, consisting of multiple B-cell epitopes, is essential for avoiding frequent false-negative results with single peptide (21) or protein antigens (24, 25). Additionally, this peptide antigen mixture mimics complex antigens while completely avoiding cross-reactive B-cell epitopes that would be present on the full proteins from which each of the peptide antigens is derived.

For detection of anti-C. trachomatis antibodies as a surrogate marker for active C. trachomatis infection, the single signal detection with Ctr Mix1 achieved 85.6% sensitivity, 25% to 44% higher than commercially available ELISAs for detection of anti-C. trachomatis IgG antibodies (Table 2). Using a composite reference standard (21, 26–28) for anti-C. trachomatis antibody status (Fig. 2), the Ctr Mix1 antigens achieved 93.9% detection sensitivity at 98% specificity (Table 3), 15.2% to 45.1% higher than the sensitivity of four commercial ELISAs (54.9 to 78.7%). Additionally, the mixed peptide assay, with 81.6% sensitivity, is also highly effective for detection of short-lived IgG3+IgA antibodies (Table 3). In analysis with likelihood ratios and predictive values, the mixed peptide assay showed a large diagnostic effect size and substantially outperformed commercial ELISAs that showed minimal diagnostic effect (Fig. 3B to D). We conclude that the higher sensitivity of the peptide assays described here is the result of the use of multiple highly reactive B-cell epitopes chosen from several immunodominant C. trachomatis proteins, while three of the commercial anti-C. trachomatis IgG ELISAs are exclusively based on OmpA antigens. The single OmpA peptide antigen from our set of C. trachomatis B-cell epitopes (peptide 1 [Table 1]) performed better than any of the commercial OmpA ELISAs (Table 5), but the Ctr Mix1 peptide antigens provided superior performance.

A single commercial assay, the GenWay ELISA, is based on the complex C. trachomatis elementary body antigen. This assay performed best among commercial ELISAs, but it still provided only moderate diagnostic effect size (Fig. 3B to D). Nonetheless, the whole C. trachomatis elementary body antigen shares a multitude of B-cell epitopes with C. pneumoniae or other *Chlamydia* spp., inherently compromising specificity by cross-reactivity with other *Chlamydia* spp. Another commercial assay, the Medac ELISA based on recombinant chlamydial LPS antigen, is commonly used for detection of anti-*Chlamydia* species antibodies when cross-reactivity is of minor concern. However, this assay performed poorly both in sensitivity and specificity, with only 61% sensitivity for detection of anti-C. trachomatis antibodies in sera from Ctr NAAT+ women (Table 2) and 11.6% false reactivity with anti-C. trachomatis antibody-negative sera (Fig. 2).

The single signal detection with a mixture of peptide antigens also achieved high assay specificity in the convenient colorimetric ELISA format, similar to the assay specificity obtained by comprehensively individually testing these C. trachomatis peptide antigens (21). Additionally, despite being highly labor saving, this ELISA format provides adequate assay sensitivity (Tables 2, 4, and 5), only marginally lower than individual peptide testing using the most sensitive chemiluminescent detection (21). Thus, the present study offers a simple ELISA with a defined mixture of synthetic antigens for anti-C. trachomatis antibody detection that has the advantage of simultaneous high sensitivity and specificity (Tables 2, 3, and 5). This format is within reach of any diagnostic laboratory, and it can be readily commercialized similar to OmpA peptide ELISAs (29–38). The single well simplicity will also allow for individual background determination in a second reaction per serum, using a blank well or a well coated with nonrelated peptides. This approach can account for high background of some sera (e.g., containing rheumatoid factor) by allowing serum-specific background subtraction, thus eliminating false-positive results from such sera.

Consistent with other studies (39), the Ctr Mix1 detected higher frequency of anti-C. trachomatis antibodies in African American individuals than in individuals of Caucasian or other origin. The Ctr Mix1 IgG ELISA detected anti-C. trachomatis IgG in 50% of Caucasian female and 20% of Caucasian male blood donors (Table 4), while the consensus of the commercial ELISAs indicated IgG reactivity in only 15% and 13%, respectively. Thus, in Caucasian females with typically low anti-C. trachomatis antibody levels (39), the Ctr Mix1 ELISA detected anti-C. trachomatis antibodies more reliably than commercial ELISAs (*P = *0.041, two-tailed Fisher exact test).

In the complete set of study sera (Fig. 4), the seroreactivity with Ctr Mix1 was 72% to 87% concordant with the commercial ELISAs. This concordance is higher than the 65% to 77% concordance that we observed in testing with individual peptides (21). The explanation for this observation is that the mixed peptide assay is less sensitive than the combined result of individual peptide assays (Table 5). Therefore, the mixed peptide assay is closer to the lower sensitivity of commercial ELISAs, thus more concordant to them. Both studies show that the majority of discordant results occurs in weakly reactive sera (Fig. 4). These discordant results are therefore due to false-negative results of commercial ELISAs rather than to false-positive results of the peptide ELISAs with proven high specificity (16–21).

Interestingly, high IgG antibodies detected in the Ctr Mix1 IgG ELISA were highly concordant with short-lived antibody isotypes IgG3 and IgA1&A2 (Fig. 4). This suggests that high IgG antibody levels tend to indicate recent exposure to C. trachomatis (33, 40, 41) and thus may be useful markers for active infection with C. trachomatis (Fig. 2). In detection of IgG, IgG3, and IgA1&IgA2 antibody isotypes, the Ctr Mix1 achieved 22.4%, 28.8%, and 29.6% higher sensitivity than Ctr Mix2 (Table 2). Thus, using only moderately reactive peptide antigens in Ctr Mix2 highly significantly reduced performance of the mixed peptide assay compared to highly reactive peptide antigens in Ctr Mix1 (Table 3).

The loss of sensitivity in the mixed peptide assay is in contrast to nearly equal sensitivity of moderate and highly reactive peptides when the peptides are individually tested and their reactivities are combined (21). In the peptide ELISA format, the total amount of 25 pmol of antigen per well has been adjusted to signal saturation, i.e., the amount of antigen exceeds the amounts of binding antibodies in sera. Under these conditions, we hypothesize that two factors explain this disproportional signal reduction of moderately reactive peptide antigens in the mixed peptide assay. (i) Mixing peptides at identical total peptide concentration per well reduces the amount of individual peptide antigens, thus reducing signals. However, given the 12-fold lower individual peptide amounts in the mixed versus individual assay, the individual antigens are still equal to or in excess of binding antibody. Therefore, we assign to antigen dilution only a minor influence on signal reduction. (ii) Binding avidity will be substantially lower if on average only a single arm of the divalent antibody can find a cognate peptide binding partner, as is the case in mixed peptide assays. In contrast, virtually all antibodies will bind with maximum avidity when both antibody arms will bind to a cognate peptide, as is the case when only identical peptide antigens are available in individual peptide assays. This argument is supported by the observation that incorporation of increasing numbers of peptides in the antigen mixture increases the reactivity difference between peptide mixtures and the maximally reactive individual peptides of the mixtures (Fig. 6). In summary, under reduced antibody binding avidity, mixtures of only moderately reactive peptide antigens (Ctr Mix2) disproportionally may experience signal reduction below assay background, while mixtures of strongly reactive peptides (Ctr Mix1) still produce signals above assay cutoff.

For seroepidemiological studies, simple assays with high specificity and sensitivity are essential for determination of past exposure and measurement of cumulative risk of C. trachomatis infection (42–45). Such tools can expedite ongoing efforts to minimize and control the burden of C. trachomatis infections in populations (46). The high performance of this multipeptide ELISA and the convenience of this method provide a strong argument for their use in trachoma control (22, 23, 47–49) and in screening for sexually transmitted infections (10, 50, 51). In conclusion, this highly specific and sensitive peptide ELISA will provide simple and yet improved C. trachomatis serology for epidemiological studies and will enhance accurate diagnosis of C. trachomatis infections, thereby mitigating sequelae from C. trachomatis infections (52–59).

## MATERIALS AND METHODS

### Sera.

For selection of anti-C. trachomatis antibody-negative sera (Fig. 1), we screened sera from 203 women and men that were collected from three cohorts: (i) 18 healthy, low-risk women who had never been diagnosed with C. trachomatis infection; (ii) 95 healthy women who were blood donors and self-reported to be free of infections (BioIVT North America & Asia Pacific, Westbury, NY); and (iii) 90 healthy men who were blood donors and self-reported to be free of infections (BioIVT). For anti-C. trachomatis antibody-positive sera, we used sera from another cohort with known exposure to C. trachomatis: (iv) sera from 125 women with C. trachomatis infection confirmed by NAAT of cervical and endometrial swab samples (20, 21). In the first cohort, all sera originated from Caucasian individuals, but in the remaining 3 cohorts the sera originated from individuals of African American, Caucasian, Hispanic, Asian, or mixed race. The age of all study subjects ranged from 18 to 38 years, with an average of 22 years.

Written consent was obtained from all serum donors. Blood donor sera were collected at BioIVT facilities located in the United States, using BioIVT Standard Operating Procedures approved by appropriate regulatory and ethics authorities. The study protocol for the remaining sera was approved by the Institutional Review Boards for Human Research of the University of Pittsburgh and the University of North Carolina (20, 21).

### Determination of anti-C. trachomatis IgG with four commercial ELISAs.

All 328 study sera were tested for anti-C. trachomatis IgG with four commercial ELISAs according to the manufacturers’ instructions as described before (21): (i) GenWay Chlamydia trachomatis IgG ELISA (GenWay Biotech, Inc., San Diego, CA), using Chlamydia trachomatis elementary bodies as antigen; (ii) Serion Chlamydia trachomatis IgG ELISA (Serion Immunologics, Würzburg, Germany), using a proprietary recombinant C. trachomatis major outer membrane protein (MOMP) segment as antigen; (iii) Savyon Chlamydia trachomatis IgG ELISA (Savyon Diagnostics Ltd., Ashdod, Israel), using proprietary OmpA species-specific peptides of different C. trachomatis serovars; and (iv) Medac plus ELISA for Chlamydia trachomatis IgG (Medac GmbH, Wedel, Germany), using a proprietary C. trachomatis-specific MOMP variable domain peptide as the antigen.

### C. trachomatis-specific peptide antigens.

Twenty-four C. trachomatis peptide antigens, identified previously with mouse and human anti-C. trachomatis antisera (16, 20), were used in this study (Table 1). The amino acid sequences of these peptide antigens are highly conserved within the major clade strains of C. trachomatis (96% to 100% sequence identity), except for OmpA and TarP peptides. Importantly, the sequences of these C. trachomatis peptides are highly evolutionarily divergent from C. pneumoniae (conservation ≤ 42% sequence identity in peptide B-cell epitopes; Table 1) and have only a marginal probability (∼0.014) of cross-reactivity with antibodies raised against non-C. trachomatis chlamydiae (16). Peptide antigens were chemically synthesized with N-terminal biotin followed by a serine-glycine-serine-glycine spacer, captured on streptavidin-coated white microtiter plates (Fisher Scientific, Roskilde, Denmark).

### Chemiluminescent and colorimetric ELISAs with C. trachomatis peptide antigens.

Primary antibodies were detected with horseradish peroxidase-conjugated secondary antibodies in ELISA as described before (20, 21). A polyclonal rabbit anti-human IgG-h+l cross-adsorbed antibody-HRP conjugate was obtained from Bethyl Laboratories, Inc., Montgomery, TX, USA (catalog no. A80-218P). Monoclonal mouse anti-human antibody conjugates were obtained from Southern Biotech, Birmingham, AL, USA: IgG1-HRP (catalog no. 9052-05), IgG3-HRP (9210-05), IgA1-HRP (B3506B4), and IgA2-HRP (9140-05).

In chemiluminescent detection (relative light unit/second, RLU), optimal concentrations of sera and conjugates were determined from titration of diluted sera and conjugates. A concentration (∼200× dilution for sera and ∼2,000× dilution for the polyclonal IgG-HRP conjugate) was chosen that provided very high signal (up to 10^5^ RLU) at low background (∼25 RLU on average) for the majority (≥95%) of the study sera. The background for each serum was determined in blank wells and in wells that were coated with nonspecific peptide antigens (20). To calculate the background corrected signal for each individual serum, 150% of the background (mean plus ∼3×CV) was subtracted from the raw signal produced by each serum.

In colorimetric detection (optical density [OD]), further optimization of the concentration for each conjugate was carried out to select a specific dilution that provided similar range of background regardless of polyclonal (IgG) or monoclonal (IgG3) or mixture of two monoclonal (IgA1 & IgA2) conjugates. For optimal assays, IgG, IgG3, and IgA1& IgA2 conjugates were diluted 32,000×, 2,000×, and 1,000×, respectively, which provided background ODs of 0.24 ± 0.11 (IgG), 0.20 ± 0.01 (IgG3), and 0.09 ± 0.02 (IgA1 & IgA2). The OD background of each serum was determined in blank wells not coated with any peptide antigen (20). The reaction with 3,3′,5,5′-tetramethylbenzidine (TMB) substrate (catalog no. 5120-0047; SeraCare) was stopped after 20 to 30 min with 0.16 M sulfuric acid. Optical density was measured at 450 nm in a Tecan Spectrafluor Plus reader. To calculate background-corrected signal for each individual serum, 108% of background (mean plus ∼CV) was subtracted from the raw signal produced by each serum. Given that background levels varied substantially between individual sera, this background correction method provided the most accurate measurement of specific signals, particularly for the 5% to 15% sera that produced high background levels.

### Preparation of peptide antigen mixtures.

For testing with all study sera, two mixtures of C. trachomatis peptide antigens were prepared as shown in Table 1. The first C. trachomatis peptide mixture (Ctr Mix1) consisted of 12 top-ranked peptide antigens (peptides 1 to 12 [Table 1]) that showed the highest reactivity score with human sera (20). The second C. trachomatis peptide mixture (Ctr Mix2) consisted of 12 moderately reactive peptide antigens (peptides 13 to 24 [Table 1]). In each mixture, peptide antigens were mixed in equimolar amounts, and each microtiter well was coated with a total of 25 pM peptides. This represents an ∼15× excess of biotinylated peptides compared to the peptide concentration required to saturate the available streptavidin binding sites for biotin.

Using the component peptides of Ctr Mix1, chemiluminescent ELISA signals produced by individual peptides tested as singlet antigen at 25 pM coating were compared to signals produced by a mixture of peptides. A set of 14 sera representing high to low reactivity were tested with 12 immunodominant C. trachomatis peptide antigens (peptides 1 to 12) and peptide mixtures of all 12, 2 × 6, or 4 × 3 constituent peptides. Thus, 12 individual peptide antigens (peptides 1 to 12) and seven antigen mixtures of peptides 1 to 12 (all 12 peptides), peptides 1 to 6 and peptides 7 to 12 (2 × 6 peptides), peptides 1 to 3, peptides 4 to 6, peptides 7 to 9, and peptides 10 to 12 (4 × 3 peptides) were tested (Fig. 6).

### Screening of 18 low-risk and 185 healthy blood donor sera for selection of negative-control sera for anti-C. trachomatis antibodies.

For selection of anti-C. trachomatis antibody-negative controls (Fig. 1), a total of 203 sera were first screened for anti-C. trachomatis antibody levels with the GenWay, Serion, Savyon, and Medac commercial ELISAs and two chemiluminescent ELISAs based on Ctr Mix1 and Ctr Mix2 (Fig. 2). For subsequent experiments and analyses, a set of 87 consensus-negative sera was selected as negative controls for anti-C. trachomatis antibodies (Fig. 2). These sera were considered negative controls based on consensus negative test results in all 4 commercial ELISAs and low or no reactivity in the 2 mixed peptide ELISAs.

### Colorimetric ELISA testing of 125 C. trachomatis-positive and 87 C. trachomatis-negative sera with Ctr Mix1 and Mix2.

A test population was created by combination of 125 women expected to have high levels of anti-C. trachomatis antibodies from active C. trachomatis infection and 87 consensus-negative sera. These 125 C. trachomatis-positive and 87 -negative sera were tested for anti-C. trachomatis IgG, IgG3, and IgA1&IgA2 antibodies with peptide mixtures Ctr Mix1 and Mix2 in colorimetric ELISAs (Fig. 2). The sensitivities of these colorimetric ELISAs were compared to those of the commercial anti-C. trachomatis IgG antibody ELISAs (Tables 2 and 3).

### Comparative assay sensitivities of mixed and individual C. trachomatis peptide antigen ELISAs.

Previously, sera from 32 blood donors with anti-C. trachomatis antibody-negative status and 125 women with Ctr NAAT-positive infection status had been tested comprehensively with 11 individual peptides (21). The performance of the mixed peptide assays (12 peptides per microtiter well [Table 5]) were compared to the anti-C. trachomatis antibody status that had been previously determined by combining individual test results of 11 of these peptides or by the single test result of the top-ranked OmpA peptide (21).

### Composite reference standards (CRS) for C. trachomatis exposure and antibody status.

All assays were evaluated by use of CRS (21, 26–28). For evaluation of C. trachomatis exposure, CRS1 (Fig. 2) was constructed by combining the 125 NAAT-positive results and 87 anti-C. trachomatis antibody test-negative results in 4 commercial ELISAs and 2 chemiluminescent Ctr Mix1 and Mix2 ELISAs. CRS2 for evaluation of C. trachomatis antibody status was derived again from these 212 (125 plus 87) sera (Fig. 2) but assuming any serum as antibody positive if the test result of any individual antibody test was positive: GenWay, Serion, Savyon, Medac anti-C. trachomatis IgG ELISAs and the IgG, IgG3, or IgA1&IgA2 ELISAs with Ctr Mix1 and Mix2 peptide antigens. For evaluation of assay sensitivities with mixed versus individual peptide antigens (Fig. 5), a CRS3 was derived from 157 (125 plus 32) sera tested by IgG, IgG3, or IgA1&IgA2 ELISAs with Ctr Mix1, or the top-ranked single OmpA peptide 1 (Table 1), and the average reactivity of 11 individual peptides 1 to 11 (Table 1).

### Assay performance evaluation by area under the ROC curves (ROC-AUC).

ROC curves were plotted as described before (21, 60; Tables 3 and 5). Following standard terminology for effect sizes (61), we termed the test discriminatory power for prediction of the C. trachomatis infection phenotype at AUCs of 0.90 to 1.00 as excellent, 0.80 to 0.90 as good, 0.70 to 0.80 as fair, 0.60 to 0.70 as poor, and 0.50 to 0.60 as failed.

### Determination of likelihood ratios and predictive values in diagnostic testing.

For evaluation of diagnostic anti-C. trachomatis antibody assay performance, positive and negative likelihood ratios (+LR and −LR) were calculated at 70% to 98% assay specificities and the corresponding sensitivity determined from ROC curves as described before (21). Based on accepted ranges of effect size (21, 61–63), the performance of an assay was deemed diagnostically useful if both +LR and −LR exceeded certain effect sizes: large effect (+LR ≥10, −LR ≤0.10), moderate effect (+LR ≥5, −LR ≤0.15), and small effect (+LR ≥2.5, −LR ≤0.25). For population prevalence-dependent evaluation of assay performance, positive and negative predictive values (PPV and NPV) of individual assays were plotted against 0% to 100% prevalence as described earlier (21, 62, 63).

### Statistical analyses.

Statistical analyses were performed, and graphical outputs were generated by the software packages Microsoft Excel 2016 (Microsoft Corporation, Redmond, Washington) or Statistica 7.1 (Statsoft, Tulsa, Oklahoma, USA). Antibody detection frequencies were compared by two-tailed Fisher exact and chi-square tests. For comparison of ROC-AUC values, the corresponding sensitivities were calculated at specificity cutoffs of 98%, 95%, 90%, 85%, and 80%. For comparison of likelihood ratios, corresponding −LR were calculated for +LR of 5, 10, 15, 20, and 25. For comparison of predictive values within given anti-C. trachomatis antibody prevalence ranges, 5 PPVs and 5 NPVs of each assay were calculated, corresponding to 5 equidistant prevalences. Means were then compared by one-tailed paired Student’s *t* test.
